# Upscaling methane fluxes from peatlands across a drainage gradient in Ireland using PlanetScope imagery and machine learning tools

**DOI:** 10.1038/s41598-023-38470-6

**Published:** 2023-07-25

**Authors:** Ruchita Ingle, Wahaj Habib, John Connolly, Mark McCorry, Stephen Barry, Matthew Saunders

**Affiliations:** 1 School of Natural Sciences, Botany Discipline, Trinity College Dublin, Dublin, Ireland; 2 School of Natural Sciences, Geography Discipline, Trinity College Dublin, Dublin, Ireland; 3 Bord na Mona, Newbridge, Ireland; 4 Water Systems and Global Change Group, Wageningen University, Wageningen, The Netherlands

**Keywords:** Ecology, Biogeochemistry, Ecology

## Abstract

Wetlands are one of the major contributors of methane (CH_4_) emissions to the atmosphere and the intensity of emissions is driven by local environmental variables and spatial heterogeneity. Peatlands are a major wetland class and there are numerous studies that provide estimates of methane emissions at chamber or eddy covariance scales, but these are not often aggregated to the site/ecosystem scale. This study provides a robust approach to map dominant vegetation communities and to use these areas to upscale methane fluxes from chamber to site scale using a simple weighted-area approach. The proposed methodology was tested at three peatlands in Ireland over a duration of 2 years. The annual vegetation maps showed an accuracy ranging from 83 to 99% for near-natural to degraded sites respectively. The upscaled fluxes were highest (2.25 and 3.80 gC m^−2^ y^−1^) at the near-natural site and the rehabilitation (0.17 and 0.31 gC m^−2^ y^−1^), degraded (0.15 and 0.27 gC m^−2^ y^−1^) site emissions were close to net-zero throughout the study duration. Overall, the easy to implement methodology proposed in this study can be applied across various landuse types to assess the impact of peatland rehabilitation on methane emissions by mapping ecological change.

## Introduction

Natural wetlands are the largest contributor of atmospheric methane which remains a major climate concern^1^. Peatlands are a type of wetlands covering only 3% of the global land area and are the largest natural terrestrial carbon store. Approximately 12% of global peatlands are degraded through anthropogenic activities and contribute to ~ 4% of the total anthropogenic greenhouse gas emissions^2^. Peatlands cover around 20% of land area in Ireland dominated by blanket bogs and raised bogs^3,4^. However, 95% of these peatlands are drained for peat extraction, agriculture and forestry^2^. Rewetting of peatlands is implemented as an effective strategy for rehabilitation of degraded peatlands by reducing their contribution to atmospheric CO_2_^5^. However, the impact of rewetting on CH_4_ dynamics is poorly understood. Additionally, there is considerable uncertainty between the top down and bottom up approach for estimation of methane emissions^1^. Quantification of methane emissions from peatlands is crucial to better understand the impacts of these ecosystems on climatic feedbacks. Further, this will assist in selecting suitable mitigation strategies for rehabilitation of degraded peatlands.

Closed chamber (CC) measurements and eddy covariance (EC) techniques are the most common field methods that are used to measure CH_4_ emissions^6–8^. EC towers typically measure trace gas fluxes with a high temporal resolution (10 Hz) across a footprint (250–3000 m radius around flux towers) and deliver a single integrated flux value across the footprint area^9,10^. This can potentially bias the flux estimation while upscaling the flux from the tower footprint to larger areas particularly for heterogeneous locations^11,12^. Peatlands are naturally heterogeneous systems with vegetation changing at a scale of less than 1 m which could significantly influence these flux measurements^13,14^. Many EC studies do not take this vegetation variation into account within tower footprints nor the impact it has on integrated flux values when reporting annual carbon (C) and greenhouse gas (GHG) budgets^12,15^. The CC technique measures methane fluxes at chamber/collar scale (~ 0.25 m^2^) with a higher spatial resolution compared to the EC as they can be distributed across the key vegetation communities present. However, the CC approach has lower temporal resolution which can introduce uncertainty in the emission estimation due to larger data gaps in addition to being labor intensive^16,17^. Vegetation communities can act as a good predictor of seasonal methane fluxes as key plant species and associated communities grow in specific locations within the ecosystem and have characteristic emission profiles^8,14,18^. Therefore, to upscale methane fluxes from the chamber to site scale requires a better understanding of the spatial distribution of the vegetation within a study area^14,19,20^.

Remote sensing is a powerful tool for mapping vegetation distribution^21–23^ and imagery can be acquired at a range of temporal and spatial scales ranging from near-Earth UAV at 5 cm, up to remotely sensed data at 5–30 m (Sentinel) or 500 m (MODIS)^8^. Erinjery et al.^24^ used a combination of Sentinel-2 (S2) MSI and Sentinel-1 (S1) data to discriminate different vegetation types in tropical rainforests with an overall accuracy of 75%. The S2 bands along with the machine learning Bagged Trees ensemble classifier have also been used to map raised bogs, turloughs, and fens in Ireland with an overall accuracy of 87%^14^. However, these studies are based on S2 imagery with 10 m resolution. PlanetScope multispectral satellite imagery (PlanetScope-0 and -1) has a spatial resolution of 3.7–4.1 m and has been used to derive accurate fine-scale vegetation maps of peatlands^22,25^. These higher resolution images can capture the spatial distribution of plant species at a finer scale unlike S2, Landsat and MODIS. They can also capture seasonal changes due to the higher temporal resolution of a 3–5 day return periods^26^. Cheng et al.^27^ compared PlanetScope and Sentinel data to assess vegetation phenology for heterogeneous landscapes in Kenya and found that the PlanetScope data were able to better capture the spatial extent of changing phenology within the landscape than the Sentinel imagery.

Machine learning techniques are widely used with remote sensing approaches for wetland mapping^14,28,29^. The Random Forest (RF) classification model^30^ based on an ensemble of several decision trees has been used in numerous global studies for mapping wetland ecology. The RF ensemble classifier was successfully used on 30,000 Landsat-8 images with the Google Earth Engine platform (GEE) to map the Canadian wetland inventory with an overall accuracy of 71%^31^. A recent study by Chimner et al.^32^ focused on mapping the vegetation distribution in the mountain meadows of Peru using multi-sensor data and RF, achieving an overall accuracy of 92%. Bhatnagar et al.^33^ compared machine learning and deep learning algorithms to map peatland vegetation communities in Ireland and identified RF as the best pixel-based machine learning classifier with a mapping accuracy of 85%. The Statistical Machine Intelligence and Learning Engine (SMILE) is a comprehensive machine learning system and its platform implements machine learning algorithms for supervised and unsupervised classification. Sujud et al.^34^ provided a comparative analysis of four machine-learning classifiers implemented in Google Earth Engine (GEE) for mapping cannabis and other crop type classifications and concluded that the SMILE Random forest (SMILE-RF) classifier outperformed all the other classifiers.

In this study, a novel approach is proposed for upscaling of methane fluxes from point scale to ecosystem scale using high-resolution satellite data and machine learning algorithm. PlanetScope imagery along with the SMILE-RF algorithm was used to create annual vegetation maps of peatland systems across a drainage gradient. These maps were used to upscale chamber-based methane fluxes for various vegetation communities using a weighted-area approach. Dominant vegetation communities were referred as ecotopes in this work although the application of ecotopes to cutaway sites has not yet been defined. The annual ecotope maps for the years 2020 and 2021were generated for three Irish midland peatlands (intact raised bog and 2 former raised bogs, now cutaway) across a drainage gradient. The drainage gradient defines the bog conditions due to degradation or management practices and this study focuses on sites degraded through recent industrial extraction, under rehabilitation and near-natural bogs^35–37^.

## Materials and methods

### Site description

**Figure 1:**
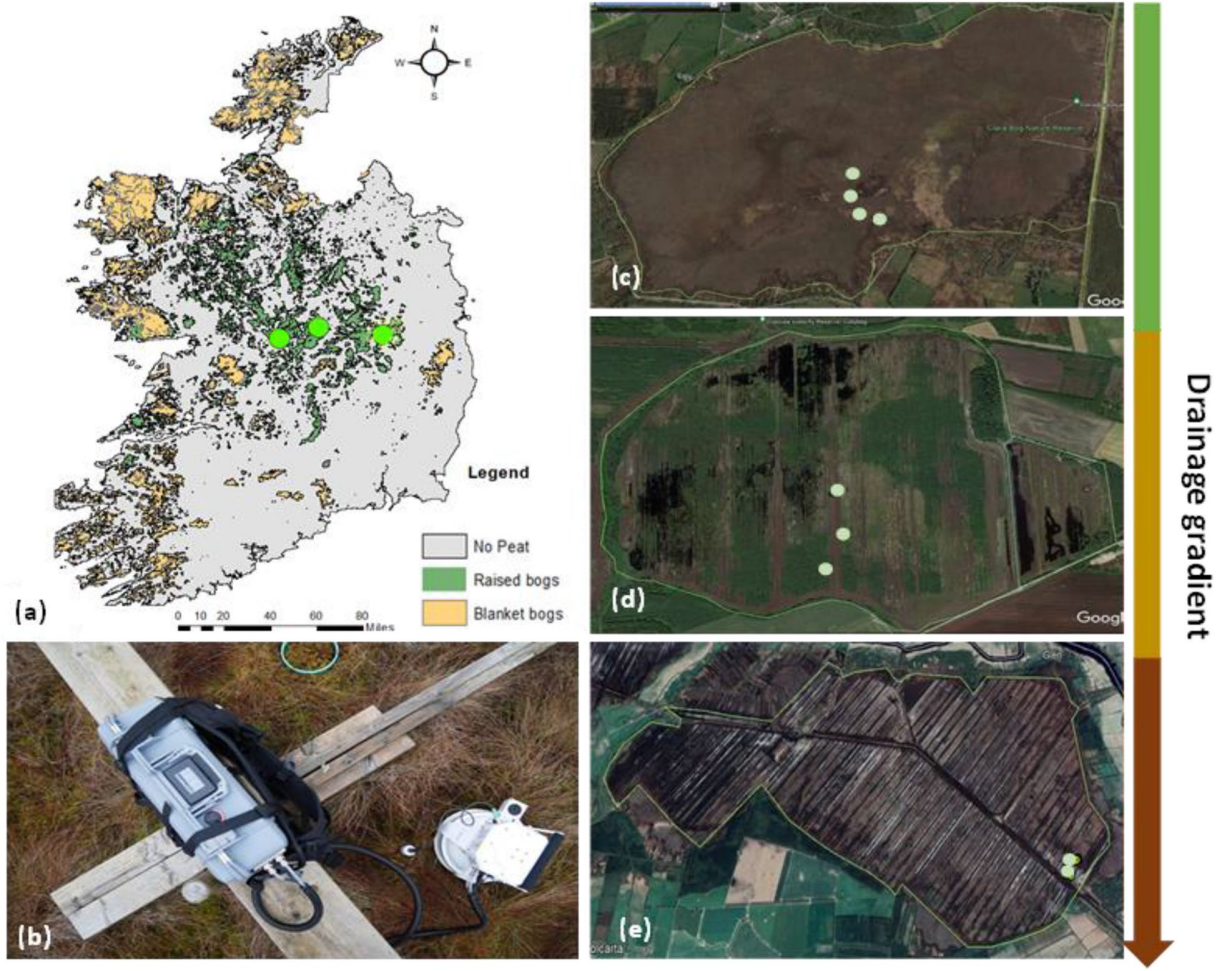
(**a**) A map of Ireland showing sites as green circles; (**b**) The chamber measurement set up with the LI-COR (LI7810) trace gas analyzer, smart chamber and PVC collar; **c** to **e** represent the sites across the drainage gradient (**c**) Clara bog (near-natural) (**d**) Lullymore (rehabilitation) (**e**) Garryduff (former industrial extraction), and the collar locations are shown as light green circles.

This study was undertaken at three raised bogs in Ireland as shown in Fig. 1a.

### Clara bog

Clara Bog is regarded as one of the largest near-natural raised bogs remaining in Ireland. It is located in County Offaly in the Irish Midlands and designated as a special area of conservation (SAC) under the European Union Habitats Directive (Council of the European Communities, 1992). The bog is bisected by a road that was put in place around 18th Century^38^ and significantly impacted the hydrology of the bog^39^. For this study, measurements were conducted on the western part of the bog which will be referred to as Clara bog in this article (Fig. 1c). Clara bog is divided into several ecotopes based on the vegetation community classification system proposed by Schouten in 2002^40^. The ecotopes are characterized by the ecological differences that are linked to the variable hydrological characteristics of the bog and range from the ecologically pristine active raised bog (ARB) to central (C), subcentral (SC), active flush (AF) and soaks, submarginal (SM), marginal (M), Inactive flush (IF) and Bog woodland (BW). The bog woodland at Clara bog has high Sphagnum cover, and has developed on deep peat. All the dominant species for each of the ecotopes are shown in Table 1. The chamber measurements were conducted fortnightly from January 2020 through December 2021. A total of 24 collars were installed at Clara Bog at the major dominant ecotopes (C, SC, SM, M) with 6 collars at each ecotope (Fig. 1c).

**Table 1:** Vegetation communities, assigned ecotopes, chamber measurements and number of collars for all the sites.

Site	Class	Ecotopes	Dominant vegetation	Chamber measurements (Yes/No)	No. of collars
Clara bog	1	Submarginal (SM)	*Narthecium ossifragum* and *Sphagnum tenellum*	Y	6
	2	Subcentral (SC)	*Sphagnum magellanicum*	Y	6
	3	Marginal (M)	*Calluna vulgaris* and *Trichophorum germanicum*	Y	6
	4	Central (C)	*Sphagnum cuspidatum*	Y	6
	5	Active flush (AF)	*Sphagnum cuspidatum, Myrica gale, Betula pubescens scrub woodland with Sphagnum palustre* and *Polytrichum commune, Molinia caerulea*	N	NA
	6	Bog woodland (BW)	*Betula pubescens, Frangula alnus, Pinus sylvestris, Pinus cortorta* and *Picea abies*	N	NA
	7	Inactive flush (IF)		N	NA
Lullymore	1	Bare peat (BP)	Bare peat	Y	4
	2	Shrub (S)	Mosaic of pioneer *Juncus, Eriophorum angustifolium*-dominated poor fen and emergent *Betula pubescens* and *Salix* spp. scrub	Y	4
	3	Bog woodland (BW)	*Betula pubescens, Pinus sylvestris, Pinus contorta* and	Y	4
	4	Open water (OW)	open water and emergent wetland vegetation	N	NA
Garryduff	1	Bare peat (BP)	Bare peat	Y	8
	2	Vegetation (V)	Mosaic of pioneer *Juncus, Eriophorum angustifolium &* Carex rostrata-dominated poor fen and emergent *Betula pubescens* and *Salix* spp. scrub	Y	8

NA: Chamber measurement were not conducted.

### Garryduff

Garryduff Bog (GD) is located in County Galway and represents a former industrial peat extraction (cutaway) site where peat production ceased in stages with complete closure in 2021 (Fig. 1e). During the extraction period it had a pumped drainage regime, and the water table was significantly lower than the surrounding area^41^. Much of the site comprises extensive areas dominated by bare peat and emerging pioneer vegetation. Parts of the cutaway now have well developed wetland and scrub vegetation. To understand the CH_4_ dynamics from natural vegetation and bare peat, the site was categorized into two main ecotopes: bare peat (BP) and vegetation (V) as shown in Table 1, and chamber measurements were conducted fortnightly from May 2020 through December 2021. At this site, 16 collars were installed with 8 at the BP and 8 at V ecotopes (Fig. 1e).

### Lullymore

Lullymore in County Kildare is also a former industrial peatland extraction site (Fig. 1d) that was previously mined but underwent rehabilitation through drain-blocking and rehabilitation on a phased basis over 15 years. Fortnightly chamber measurements were conducted from May 2020 through December 2021 at three dominant vegetation communities (Table 1) as follows: bare peat (BP), shrub (S), and bog woodland (BW) based on a Bord na Mona habitat map^42^. Bog woodland at Lullymore is on shallow peat < 1 m deep and is defined by relatively dry peat conditions with no *Sphagnum*. All the dominant vegetation species are shown in Table 1. At the Lullymore site, 12 collars were installed with 4 collars each at the BP, S and BW (Fig. 1d).

### In-situ measurements

Methane fluxes were measured using the chamber technique^20,43^ at Garryduff and Lullymore for 19 months and 24 months at Clara bog. A laser-based analyzer (LI-7810, CO_2_/CH_4_/H_2_O Trace Gas Analyzer; LI-COR Biosciences) in conjunction with the LI-COR Smart Chamber portable unit (LI-8200-01S, Smart Chamber; LI-COR Biosciences) system was used to measure CH_4_ concentrations (Fig. 1b). The methane flux was calculated using curvilinear function in the LICOR SoilFluxPro software version 5.2. In addition to the measurement of soil gas fluxes, an auxiliary HydraProbe (Stevens Water Monitoring System) provided simultaneous monitoring of soil temperature, moisture, and electrical conductivity. Methane measurements were recorded fortnightly except during the COVID-19 lockdown when travel across Ireland was restricted. The average daily flux values from all the collars were used to estimate average annual methane flux for each ecotope and the units were converted to gC m^−2^ y^−1^.

### Satellite data

The PlanetScope data was acquired by the Planet labs Inc., with their low-cost constellation of satellites. The data has a spatial resolution of 3.70–4.10 m which is resampled and distributed by two sensors i.e., PlanetScope–0 and PlanetScope-1 to achieve 3 m spatial resolution^44^. The imagery was atmospherically corrected using the 6S model (Second Simulation of a Satellite Signal in the Solar Spectrum). The data was accessed and downloaded using the Planet Labs ArcGIS Pro add-in. The imagery consists of four bands: Red (590–682 nm), Green (500–585 nm), Blue (455–517 nm) and NIR (780–880 nm), and all the four bands were used for the analysis in this study. The images were collected in 2020 and 2021 for all the three sites. A cloud filter of less than 10% was used and a total of 35 images were collected for Clara bog, 20 for Garryduff and 19 for the Lullymore site. There were no cloud free images available in the summer at Clara bog and Garryduff nor for winter months at Lullymore in 2020. Three Vegetation Indices: normalized difference vegetation index (NDVI), enhanced vegetation Index (EVI) and green normalized difference vegetation index (GNDVI) were also calculated and added to the RF classification to improve the classification results.

### Ecotope mapping

**Figure 2:**
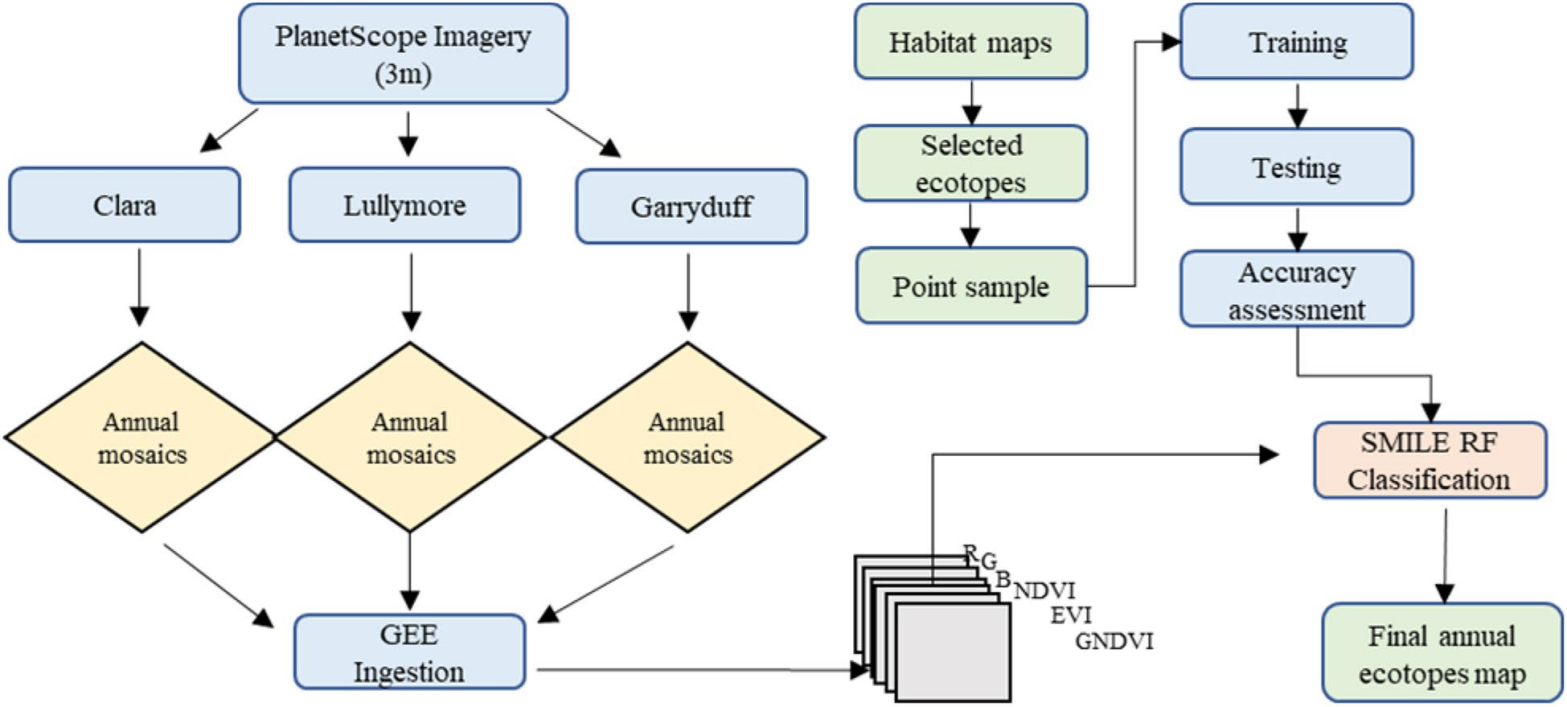
Image classification workflow for annual ecotope map generation in GEE.

**Figure 3:**
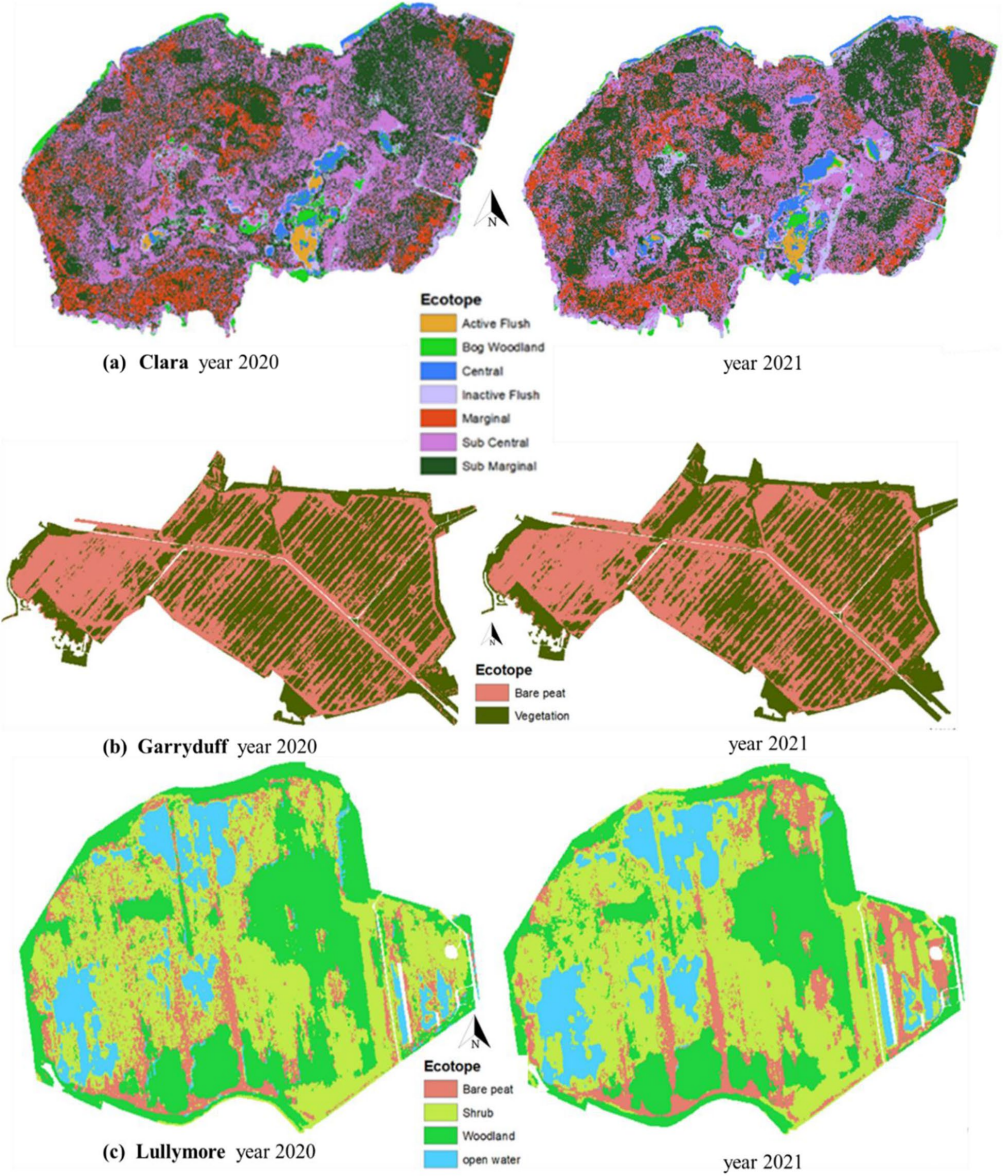
Annual ecotope maps for years 2020 and 2021 at (**a**) Clara bog (**b**) Garryduff (**c**) Lullymore site.

The PlanetScope data was used to map the ecotopes at the three study sites. A machine learning-based RF image classification SMILE technique was used in the Google Earth Engine (GEE) platform to perform pixel-based image classification (Fig. 2). All the available satellite images were ingested into GEE to create annual ecotope maps for 2020 and 2021. A combination of habitat maps provided by Bord na Mona (Lullymore and Garryduff sites) and National Parks and Wildlife Service (Clara bog) along with high-resolution Google earth imagery^45–47^ were used to train and test the models with a split of 70:30. A total number of 144 polygon samples (20,578 pixels) were defined based on a random distribution across all the seven ecotopes at Clara bog. A similar approach was implemented at Lullymore with a total number of 166 polygons (30,847 pixels) with random distribution over the four ecotopes. For the Garryduff site, a total number of 192 polygons (10,922 pixels) were created using a random distribution across the two ecotopes. Furthermore, an accuracy assessment of the results was conducted and a confusion matrix with Overall validation Accuracy (OA), Producer Accuracy (PA) and user accuracy (UA) was developed (Tables 2, 3 and 4). Kappa coefficient was not reported for image classification as recommended by Foody^48^ due to its limitation in the accuracy assessment of thematic maps. The annual ecotope maps generated for all the three sites for the years 2020 and 2021 are shown in Fig. 3. The area of each ecotope is estimated from the annual ecotope maps for the relevant year and was used for upscaling methane fluxes outlined in Eq. (1).

**Table 2:** Validation accuracy assessment based on number of pixels at Clara bog for the year 2020 and 2021.

	SM	SC	M	C	AF	BW	IF	Total	PA (%)
Clara 2020									
SM	**2099**	246	88	0	0	2	0	2435	86
SC	285	**1751**	53	0	0	3	4	2096	83
M	116	89	**564**	0	0	0	0	769	73
C	0	3	0	**211**	9	15	6	244	86
AF	0	1	0	11	**214**	6	9	241	89
BW	1	4	0	10	1	**216**	5	237	91
IF	21	40	1	3	7	7	**120**	199	60
Total	2522	2134	706	235	231	249	144	**6221**	**OA** **83%**
UA(%)	83	82	80	90	93	87	83		
Clara 2021									
SM	**2136**	228	42	0	0	0	1	2407	89
SC	270	**1748**	87	0	0	0	5	2110	83
M	123	120	**542**	0	0	0	0	785	69
C	0	1	0	**203**	7	1	0	212	96
AF	0	0	0	6	**204**	2	3	215	95
BW	2	3	2	1	2	**227**	7	244	93
IF	18	14	1	1	7	7	**166**	214	78
Total	2549	2114	674	211	220	237	182	**6187**	**OA** **84%**
UA(%)	84	83	80	96	93	96	91		

Significant values are in [bold].

**Table 3:** Validation accuracy assessment based on number of pixels at Garryduff for the year 2020 and 2021.

	BP	V	Total	PA (%)
GD 2020				
BP	**4182**	19	4201	99
V	8	**5067**	5075	100
Total	4190	5086	**9276**	**OA** **99%**
UA (%)	99.81	99.63		
GD 2021				
BP	**4095**	30	4125	99
V	17	**5056**	5073	100
Total	4112	5086	**9198**	**OA** **99%**
UA (%)	100	99		

Significant values are in [bold].

**Table 4:** Validation accuracy assessment based on number of pixels at Lullymore for the year 2020 and 2021.

	BP	S	BG	OW	Total	PA (%)
LM 2020						
BP	**167**	17	1	0	185	90
S	10	**380**	7	1	398	95
BW	2	4	**1766**	0	1772	100
OW	0	0	0	**261**	261	100
Total	179	401	1774	262	**2616**	**OA** **98%**
UA (%)	93	95	99	100		
LM 2021						
BP	**154**	3	2	0	159	97
S	10	**390**	0	0	400	97
BW	0	0	**1771**	0	1771	100
OW	0	2	1	**263**	266	99
Total	164	395	1774	263	**2596**	**OA** **99%**
UA (%)	94	99	100	100		

Significant values are in [bold].

### Upscaling

A simple area-weighted average method has been used previously in several studies and found to be an effective and simple upscaling approach^23,49^. The same approach is used here to upscale the chamber CH_4_ fluxes, using Eq. (1) below:

1 $${\mathrm{Flux}}_{\mathrm{upscaled}}=\frac{\mathrm{mean\,chamber\, derived\, flux }*\mathrm{ ecotope\, area}}{\mathrm{Total\, area}}$$

## Results

### Spectral profiles

The spectral signatures of each ecotope at the three sites were plotted (see Supplementary Figure S1 online). For Clara bog, the active flush ecotope showed the highest reflectance values followed by the central ecotope, bog woodland, inactive flush, submarginal and subcentral ecotopes, while the marginal ecotopes had the lowest reflectance values for most of the bands. The differences in reflectance between the central and marginal ecotope is notable. The submarginal, subcentral and marginal ecotopes have similar reflectance values in RGB and NIR wavelengths (see Supplementary Figure S1 online). The vegetation ecotope at the Garryduff site showed highest reflectance value and bare peat showed lowest reflectance values (see Supplementary Figure S1 online). At the Lullymore site, the reflectance values of shrub and open water were noticeably different and Bog woodland showed highest reflectance value for the NIR wavelength (see Supplementary Figure S1 online). All the ecotopes, except the submarginal, subcentral and marginal ecotopes at Clara bog can be distinguished based on their spectral signatures (see Supplementary Figure S1 online).

### Annual ecotope maps

#### Clara Bog

The annual ecotope maps derived from the PlanetScope imagery and SMILE-RF model showed variability between years (Fig. 3a). The submarginal, subcentral, marginal and bog woodland ecotopes showed decline in area of around 2%, 2%, 5% and 35%, respectively in 2021 as compared to 2020 (Table 5). However, the central, active flush and inactive flush ecotope areas were shown to increase by 53%, 20% and 56% in 2021 when compared to 2020.

**Table 5:** Upscaled annual methane fluxes at all locations.

Site	Ecotope	Area based on annual ecotope map 2020 (Ha)	Area covered by ecotopes in 2020 (%)	Upscaled methane flux in 2020 (gC m^−2^ y^−1^)	Upscaled methane flux in 2020 at site scale (gC m^−2^ y^−1^)	Area based on annual ecotope map 2021 (Ha)	Area covered by ecotopes in 2021 (%)	Upscaled methane flux in 2021 (gCm^−2^ y^−1^)	Upscaled methane flux in 2021 at site scale (gCm^−2^ y^−1^)
Clara bog	SM	99.42	93	0.60	**2.25**	97.1	91	1.06	**3.80**
	SC	91.92		1.34		89.8		2.16	
	M	32.32		0.19		30.62		0.30	
	C	5.03		0.12		7.70		0.28	
Lullymore	BP	16.06	89	0.00	**0.17**	18.92	88	0.00	**0.31**
	S	58.43		0.51		57.02		0.66	
	BW	42.84		− 0.34		41.06		− 0.35	
Garryduff	BP	456.47	100	0.01	**0.15**	466.82	100	0.02	**0.27**
	V	480.83		0.14		470.49		0.25	

#### Accuracy assessment

In the 2020 assessment (Table 2), the highest producer’s accuracy (PA) of 91% was seen for the bog woodland and the lowest PA (60%) was observed for the inactive flush which was misclassified as submarginal and subcentral. The submarginal, subcentral, central and marginal ecotopes were classified with PA ranging from 73 to 89%. The marginal ecotope had a PA of 73% and it was mostly misclassified as subcentral and submarginal. The user accuracy (UA) ranged from 80 to 93% with lowest for the marginal ecotope and highest for active flush ecotope. In 2021, the highest PA was acquired at the central ecotope (96.%) followed by active flush (95%) and bog woodland (93%) as shown in Table 2. The marginal ecotope was misclassified as submarginal and subcentral with PA of 69% and inactive flush was misidentified as submarginal and subcentral with PA of 78%. The UA ranged from 80 to 96% with highest accuracy for central ecotope (96%) and lowest for the marginal ecotope (80%). During both the years, the active peat forming ecotopes such as the central and subcentral areas were identified with PA and UA ranging from 82 to 96% for all the seasons. The overall validation accuracy (OA) of 83% was observed for 2020 and 84% for 2021 (Table 2). Table 5 shows total estimated area in hectares for each ecotope for the years 2020 and 2021.

#### Garryduff

The annual ecotope maps are shown in Fig. 3b and the total area for each ecotope for 2020 and 2021 is shown in Table 5. Here, an OA of 99% was observed for both the years with PA and UA of almost 100% (Table 3). The vegetation and bare peat were mapped very well at this site for both the years.

#### Lullymore

The annual ecotope maps are shown in Fig. 3c and the total estimated area of each ecotope for 2020 and 2021 is shown in Table 5. The bare peat and open water ecotopes showed an increase in area of 17% and 2% in 2021 compared to 2020. However, the shrub and bog woodland ecotopes showed a decrease of 2% and 4% in area, in 2021 vs 2020 respectively. An OA of 98% was observed for 2020 with the PA ranging from 90 to 100% (Table 4). The highest PA was seen for open water followed by bog woodland, shrub and bare peat. The UA is also highest for open water followed by bog woodland, shrub and bare peat. For 2021, an OA of 99% was observed. The PA of bare peat (97%) and shrub (97%) was higher in 2021 as compared to 2020 and open water (99%) PA was lower in 2021 against 2020.

### Upscaled methane flux

Overall, the non-weighted average annual methane flux values measured at the various ecotopes on Clara bog in 2021 were higher than in 2020. The average annual methane flux values ranged from 1.44 to 5.75 gC m^−2^ y^−1^ in 2020 and from 2.40 to 8.89 gC m^−2^ y^−1^ in 2021, with the highest fluxes measured at the central ecotope and lowest at the marginal ecotope during the study duration (see Supplementary Figure S2 online). The (area-weighted) upscaled methane flux values based on Eq. 1 varied between 0.12 and 2.16 gC m^−2^ y^−1^ at Clara bog (Table 5). The lowest upscaled flux was observed at the central ecotope in 2020 followed by marginal, submarginal and subcentral. The upscaled methane flux followed similar trend in 2021 as shown in Table 5. At the Lullymore site, the average annual methane fluxes ranged from − 1.08 to 1.14 gC m^−2^ y^−1^ in 2020 and − 1.10 to 1.55 gC m^−2^ y^−1^ in 2021. The bog woodland soil was observed to be assimilating methane throughout the study duration and methane emissions from bare peat were close to zero. The upscaled methane fluxes at the ecotope and site scale are shown in Table 5. The average annual methane fluxes at Garryduff ranged between 0 to 0.27 gC m^−2^ y^−1^ in 2020 and 0 to 0.53 gC m^−2^ y^−1^ in 2021, and the upscaled fluxes are shown in Table 5. As all the ecotopes assessed represent > 88% of the total study area at each site, ecosystem scale emissions are also provided in Table 5. Clara bog had the highest methane emissions followed by Lullymore and Garryduff where both the sites were showed similar emission scenarios.

## Discussion

### Ecotope mapping

Mapping peatland vegetation is very challenging due to the complex heterogeneity which can change over a few meters^18,21,23^. This study presents a novel, yet robust attempt to assess the potential of using PlanetScope satellite imagery with RF machine learning algorithm (SMILE) to create annual ecotopes map for a variety of peatland ecosystems. The mapping methodology developed here was successfully validated at sites across drainage gradient with an overall validation accuracy of above 83%. Most temperate raised bogs are located in geographical areas with significant cloud cover which makes it challenging to acquire satellite imagery for similar dates as chamber measurements^14,22,25^. The satellite imagery acquired for this study was selected to be as close as possible to the site level flux measurement dates. Summer and winter imagery were not available for the year of 2020, so this study focused on developing annual ecotope maps for area estimation instead of adopting a seasonal perspective. The accuracy assessments were slightly lower at Clara bog as compared to other two sites due to greater spatial heterogeneity in vegetation across the site. Additionally, the producer and user accuracy was substantially increased due to inclusion of summer imagery in 2021 for central, inactive flush and bog woodland ecotopes as compared to 2020 (Table 2). The user accuracy of subcentral, marginal and submarginal ecotopes is acceptable (> 80%) although lower than other ecotopes at Clara bog due to their similar spectral signatures (see Supplementary Figure S1 online). In future, these challenges can be addressed by including a longer dataset and applying a multi-sensor approach while training and testing the models. It is worth highlighting that the producers accuracy at the most important active raised bog (ARB) forming ecotopes at Clara has been improved from 66 to 83% for subcentral ecotope and from 86 to 96% for central ecotope as compared to the nested drone-satellite approach by Bhatnagar et al.^33^. Not opting for the majority voting approach which provide pixel value based on majority of nearby pixels values and PlanetScope data could be responsible for achieving higher accuracy in this study. Overall, the methodology suggested here was able to map the dominant vegetation communities across all the sites with an overall accuracy of ≥ 83%. This approach can be further developed and applied to more accurately define the key ecotopes on cutaway peatlands and to map the impact of rewetting and rehabilitation on ecological change as well as the long-term monitoring of peatlands.

### Upscaled flux chamber measurements

The average annual methane fluxes measured at Clara bog(near-natural) during this study period were in the range with the chamber measurements previously conducted at Clara bog from 2015 to 2017^50^. The central ecotope average annual methane flux in 2020 (5.75 gC m^−2^ y^−1^) and 2021 (8.89 gC m^−2^ y^−1^) were in a similar range as those measured between 2015 and 2017 (7.95 ± 4.05 gC m^−2^ y^−1^) ^50^. Additionally, the measured methane flux at the subcentral ecotope in 2020 (3.60 gC m^−2^ y^−1^) and 2021 (5.93 gC m^−2^ y^−1^) were in the range with the 2015–2017 data for Clara bog (6.52 ± 2.10 gC m^−2^ y^−1^)^50^. Similarly, the annual methane flux at the submarginal ecotope in 2020 (1.48 gC m^−2^ y^−1^) and 2021 (2.70 gC m^−2^ y^−1^) were also in the range with the 2015–2017 data (3.37 ± 1.18 gC m^−2^ y^−1^)^50^. The flux values from the marginal ecotope in 2020 (1.44 gC m^−2^ y^−1^) and 2021 (2.44 gC m^−2^ y^−1^) were slightly higher than the 2015–2017 range (0.82 ± 0.37 gC m^−2^ y^−1^)^50^. The upscaled, site scale methane flux at Clara bog (2.25 gC m^−2^ y^−1^ and 3.80 gC m^−2^ y^−1^) were slightly lower than the median methane flux range (3.30–6.30 gC m^−2^ y^−1^) from northern natural peatlands^51^. Additionally, the upscaled site scale methane flux at Clara bog for 2020 (2.25 gC m^−2^ y^−1^) and 2021 (3.80 gC m^−2^y^−1^) were in the range of Tier 2 emission factors (EF) (2.20–8.70 gC m^−2^ y^−1^) proposed for near-natural peatlands in Ireland by Aitova et al.^52^. However, these upscaled fluxes were lower than the Tier 1 EF (0.30–44.50 gC m^−2^ y^−1^) based on the IPCC Wetland Supplement^53^ The average annual methane flux measured at Garryduff site (degraded) were in the range with other similar bare peat sites in Ireland, with the flux values showing net zero methane emissions^54^. The upscaled methane flux at Garryduff for 2020 (0.15 gC m^−2^y^−1^) and 2021 (0.27 gC m^−2^y^−1^) were in the range of Tier 1 EF (0.12–0.83 gC m^−2^ y^−1^) as proposed by IPCC Wetland Supplement for industrial cutaway nutrient poor sites^52^. A range of under rehabilitation sites from Ireland and 16 sites from northern peatlands (latitude 40° to 70°N) showed a similar average annual methane emissions trend to the Lullymore(under rehabilitation) site with nearly zero emissions from bare peat, and small emissions from shrub areas with assimilation from bog woodland soils^51,54^. Additionally, the upscaled methane flux at Lullymore for 2020 (0.17 gC m^−2^y^−1^) and 2021 (0.31 gC m^−2^y^−1^) were lower than the Tier 1 EF (2.24–8.69 gC m^−2^ y^−1^) as proposed by IPCC wetland supplement^52^. One of the reasons for this could be due to inclusion of near natural, rewetted peat extraction sites and rewetted grassland sites into one category for providing Tier 1 EF^52^. Although degraded, near-natural and under-rehabilitation peatlands are low methane emitters as compared to rewetted agricultural bogs, understanding methane dynamics of these ecosystems can assist in optimizing the trade-off between CH_4_ and CO_2_ emissions for effective management of these ecosystems^55^.

This study is the first attempt to use high resolution satellite imagery and machine learning to upscale chamber fluxes in Ireland. There is potential to implement the framework developed in this study to upscale chamber and EC measurements across other types of peatlands such as blanket bogs, fens and turloughs which are heterogenous and complex to map. Additionally, this approach can be useful to upscale remote sensing derived carbon fluxes across various scales^56^. Site scale methane emissions from bare peat and natural vegetation can be useful to analyze the before and after impacts of rewetting and rehabilitation. Similarly, methane emissions from under rehabilitation sites such as Lullymore provide us with information on emissions from various dominant vegetation communities and can act as a wider indicator for effective rehabilitation measures. In order to improve estimates of methane flux variations over time, an understanding of the phenological dynamics of vegetation communities and their spatial variation could be better than simply using measurements from one point in time^57^. The use of automatic chambers could potentially bridge the gap between EC tower measurements and spatial chamber measurements as they provide detailed flux measurements with a better temporal resolution. Although automated chambers could improve the point-budget, upscaling from point to ecosystem may introduce higher uncertainty for low-emitting sites such as Garryduff and Lullymore. Additionally, inclusion of key drivers such as water table depth and soil moisture while training the model could help improve the accuracy further.

## Conclusion

This study tested the applicability of high-resolution multispectral satellite imagery and a mapping technique with machine learning algorithms to provide fine-scale vegetation maps. Furthermore, these maps were used to upscale chamber-based methane fluxes at peatland sites in Ireland across a drainage gradient. Our results provide evidence that upscaling is successful if the dominant vegetation communities are appropriately mapped. Results from this study highlight the complex spatial heterogeneity of peatlands and their impact on methane fluxes, and emphasizes the need to integrate dominant plant species into methane budget models with wider upscaling approaches. Overall, the novel use of remote sensing products and machine learning algorithms will continue to provide valuable data for improving the upscaling approach by understanding impacts of key environmental drivers and vegetation dynamics on methane emissions to further inform effective rehabilitation measures.

## Supplementary Information

Supplementary Figures.

## Data Availability

Data used in the study is available from the corresponding author on a reasonable request.
