# Supplementary material for: Upscaling methane fluxes from peatlands across a drainage gradient in Ireland using PlanetScope imagery and machine learning tools
Source: Sci Rep. 2023 Jul 25;13:11997. doi: 10.1038/s41598-023-38470-6 (PMC10368722; doi:10.1038/s41598-023-38470-6)
Supplement: Supplementary file 1 — Supplementary Figures. [file 41598_2023_38470_MOESM1_ESM.pdf]

Upscaling methane fluxes from peatlands across a drainage gradient in Ireland using PlanetScope imagery and machine learning tools

(Supplementary Material)

Ingle Ruchita [a, d] *, Habib Wahaj [b], Connolly John [b], Mark McCorry [c], Stephen Barry [c] and Saunders Matthew [a]

a) Trinity College Dublin, School of Natural Sciences, Botany Discipline, Dublin, Ireland

b) Trinity College Dublin, School of Natural Sciences, Geography Discipline, Dublin, Ireland

c) Bord na Mona, Leabeg, Co. Offaly, Ireland.

d) Wageningen University, Water Systems and Global change, Wageningen, The Netherlands

Supplementary Figure for section 3.1

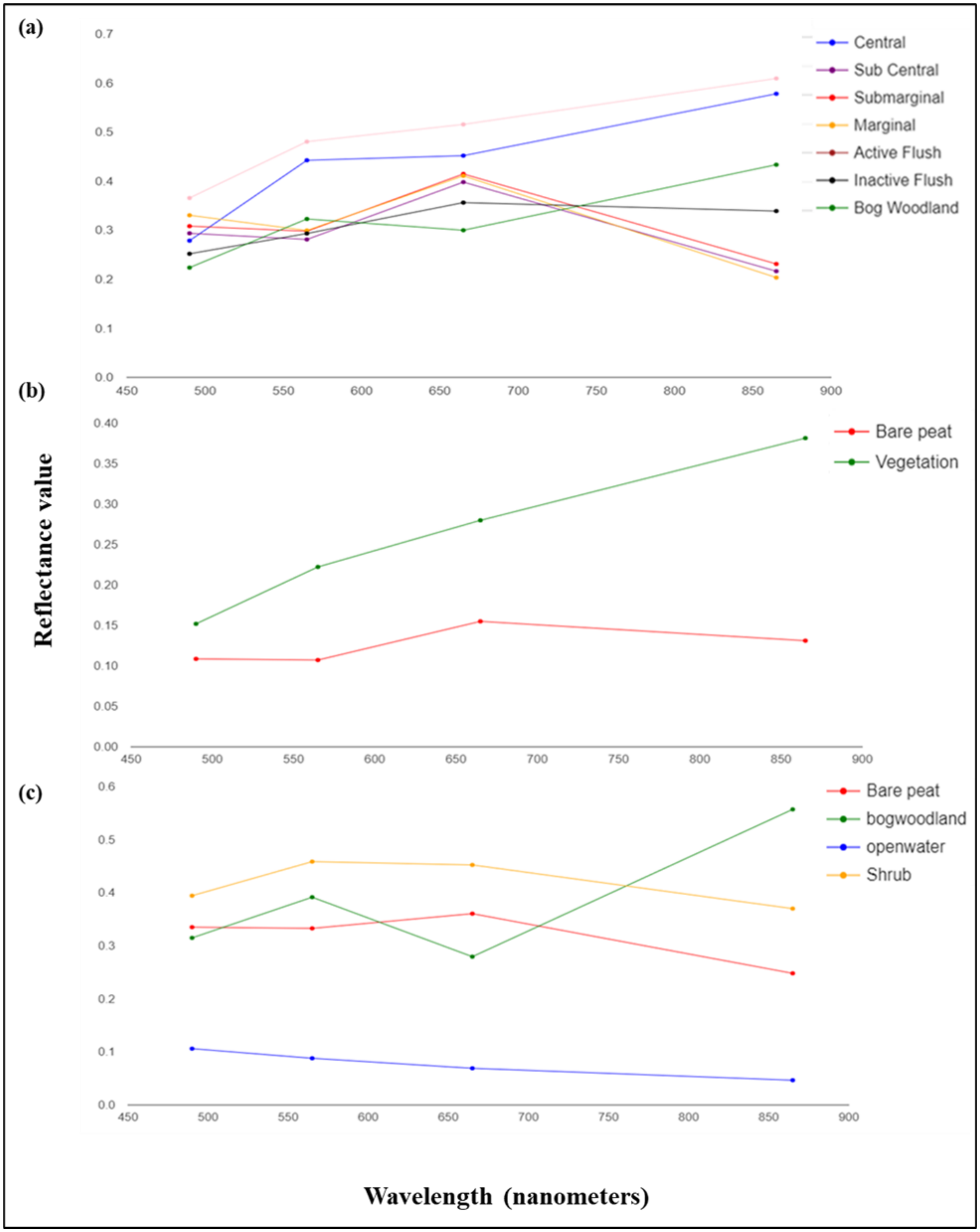

Supplementary Figure S1: A plot showing the reflectance values across various wavelengths for the ecotopes at (a) Clara (b) Garryduff (c) Lullymore

Supplementary Figure for section 3.3

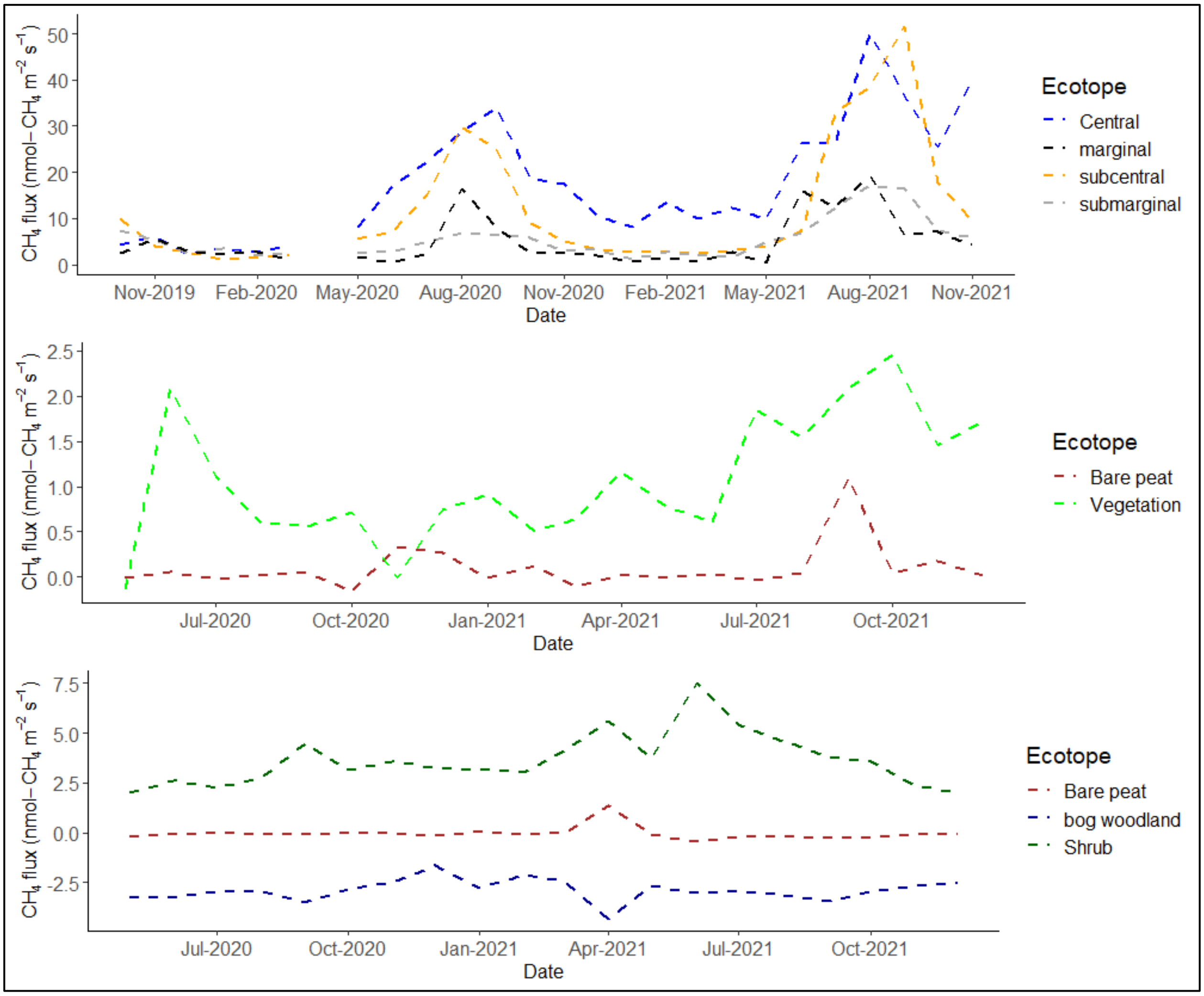

Supplementary Figure S2: Average methane flux measurements throughout the study duration at (a) Clara (b) Garryduff (c) Lullymore
